# Wettability and sound absorption of graphene oxide doped polymer hydrogel

**DOI:** 10.1038/s41598-021-95641-z

**Published:** 2021-08-05

**Authors:** A. Khosrozadeh, R. Rasuli, H. Hamzeloopak, Y. Abedini

**Affiliations:** Department of Physics, Faculty of Science, University of Zanjan, Zanjan, Iran

**Keywords:** Applied physics, Nanoscale materials

## Abstract

In this paper, we introduce a nanocomposite as a humidity-sensitive sound absorber. The nanocomposites were prepared using hydrogel polymer (HP) as a matrix and graphene oxide (GO) as a filler. Results show that the surface energy of the nanocomposite is 58.4 mJ m^−2^, and GO sheets increase the nanocomposite porosity from 2.6716 cm^2^ g^−1^ (for HP) up to 3.246 cm^2^ g^−1^. In addition, the diameter of nanocomposite pores is 8.5202 nm lower than that of HP (10.274 nm). To study the effect of humidity on the sound absorption, we exposed them to moisture for 30 and 60 min and then measured sound absorption. Results show an absorption peak for the HP at 1022 Hz with an attenuation value of 30%, while the nanocomposite shows two main peaks around 1898 and 3300 Hz. In addition, results show that sound absorption peaks shift to higher frequencies according to humidification time.

## Introduction

Sound absorbers have attracted remarkable attention in recent years due to their promising application in industry and life^1–3^. Sound-based devices can be divided into two categories with amorphous and crystal structures^4,5^. Phonon crystals have been widely studied to fabricate sound-based devices^6^. For instance, Xue-Feng et al*.* designed and simulated acoustic crystals to transfer the sound in one direction. Such a system is made from square steel blocks and can control the frequency of transmitted sound by mechanical rotation of the blocks^7^. In addition, by creating defects in a two-dimensional cylindrical grid immersed in water, one can conduct the sound waves and control their frequency^8^. In addition, topological acoustic insulators are interesting due to their applications as sound absorbers^9,10^.

Among various sound absorber materials, nanocomposites are promising for sound-based devices^11^. Typically, porous materials are applied as a sound absorber, and many studies have been performed to understand the absorption mechanism of these materials^12^. The propagation of sound is affected by environmental conditions, and sound absorption is dependent on ambient humidity and temperature^13,14^.

The low-cost and biocompatibility of hydrogel polymers introduce them as a promising candidate for sound absorbing in the transportation and construction industries^15^. Hydrogels are hydrophilic polymer with cross-linking and swollen ability, which retains large amounts of water. The moisture absorption of hydrogels by hydrophilic functional groups and their dissolution resistance are desirable to fabricate efficient sound absorbers^16^. The swelling behavior has a significant effect on the physical and mechanical properties of the final hydrogel, and environmental humidity and temperature affect the hydrogel-based sound absorber. Adding GO to the HP can improve its mechanical, thermal, hydrophilicity, and swelling properties. In addition, the properties of HP can be improved by varying concentrations of cross-linking agents in nano-composites^11,12^. Surrounding the reinforcing phase by hydrogel can improve sound absorption and increase the dissipation coefficient^6,11^. The size of the pores in a sound absorber determines the frequency of sound absorption^17–19^. Controlling the sound in resonant cavities, acoustic diodes, thermoelectric is the subject of recent researches^20,21^, and sound waves can be controlled by phonon crystals^20^. To achieve an impedance-tunable sound absorber, one should make it sensitive to the external actuator. Synthesizing low-cost and efficient tunable sound absorbers is a challenge in acoustic science^22,23^. As reported, reinforcing phases such as GO can be used as a reinforcing phase of composite to fabricate an efficient electromagnetic and sound wave absorber^11,24,25^.

In this paper, we used HP as a base polymer to fabricate humidity-sensitive sound absorber coating. GO was used to modify its surface energy and sound absorption, and the sound absorption of the prepared samples was measured after exposure to moisture at different times. In addition, the sound absorption peak of HP shifts from 1022 Hz to 2000 and 3538 Hz after humidification, showing hydrogen bond formation due to absorbed moisture that reinforces the polymer. However, for HGO samples, the frequency of sound absorption peak is 1898 Hz, which changes to higher values by humidification for 30 and 60 min, respectively. This paper is organized as follows: in the materials and method section, we present the composite preparation and characterization methods. In the results and discussion section, we discuss the prepared sample composition and porosity. Finally, we present sound absorption results and discuss the effect of humidity on sound absorption of the prepared samples.

## Materials and methods

### GO synthesis

GO was synthesized using the improved hummers method as reported previously by us^11,26^. In a typical method, we dissolved 0.1 g of graphite with 15 ml of sulfuric acid (98%) and then stirred for 15 min at 25 °C. Afterward, we added 0.3 g of KMnO_4_ to the solution and placed it in a water/ice bath for 5 min. Afterward, KMnO_4_ was removed by H_2_O_2_ at 40 °C, and then graphite oxide was exfoliated by ultra-sonication. Finally, the solution was placed in a centrifuge with a speed of 1200 RPM to separate GO from graphite oxide, as reported previously by us^11^.

### Composite synthesis

First, we milled the 500 g of potassium polyacrylate (from Atieh Energy Company) for 60 min to obtain a powder. The resulting powder dissolved in 100 ml of deionized water and then stirred for 30 min at 35 °C. Afterward, we added 5 ml of hydrochloric acid (37%) with 100 ml of deionized water to the resulting mixture. Finally, we dried the resulting solution in an oven at 70 °C for 12 h. To dope the GO in HP toward obtaining the most humidity-sensitive sample, we added 20 ml of GO solution with a concentration of 2 g l^−1^ to the primary solution and then dispersed it for 30 min by sonication. The prepared polymers for measuring the sound absorption were made using a laser cutting machine of 3 cm in diameter and 1 cm in thickness. The sound absorption measurement conditions were performed according to Table 1.

**Table 1:** Environmental conditions for measuring sound absorption.

Atmospheric pressure (Pa)	88,310
Temperature (°C)	27
Humidity (%)	38
Sound speed (m s^−1^)	347
Air density (kg m^−2^)	1

### Characterization

FTIR spectroscopy was performed by Thermo scientific model Nicolet iS10. Field emission scanning electron microscopy (FESEM) and X-ray diffraction spectroscopy (EDX) was done by MIRA3 from TSCAN Company. Brunauer Emmett Teller’s (BET) experiment was performed using a Belsorp mini II from Microtrac Bel Corp. The weight of HGO for BET analysis was 0.0586 and 0.0547 g. Impedance Tube was utilized from Manufacturer of BSWA Technology Company Model: BSWA, SW477 + SW422. Raman spectroscopy was performed using Handheld Raman Analyzer (Firstguard) from Rigaku by an exciting wavelength of 1064 nm. Thermogravimetric analysis (TGA) was done by TGA-DTA (Q600) from TA Company, and Transmission Electron Microscopy (TEM) images were recorded by Philips EM208S 100 kV.

## Results and discussions

### Composite characterization

**Figure 1:**
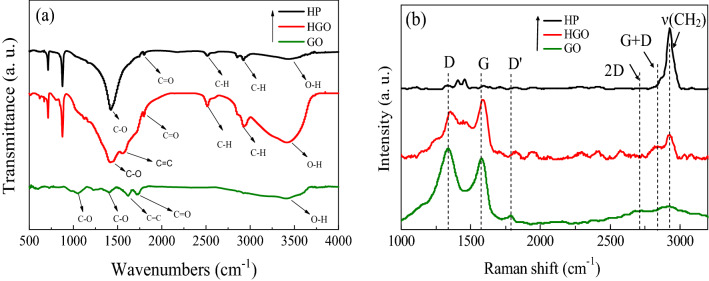
(**a**) Raman and (**b**) FTIR spectrum of GO, HP, and HGO.

FTIR is a powerful method to determine functional groups of the prepared nanocomposite^27^. Figure 1a shows the FTIR spectra for GO, HP, and an HGO. In a typical FTIR spectrum for GO, the peaks at 3405, 1622, and 1732 cm^−1^ are attributed to O–H, C=O, and C=C stretching modes. In addition, the peak observed at 1053, and 1395 cm^−1^ are attributed to the C–O stretching and bending modes. The peak at 2923 cm^−1^ is related to the C–H, and two peaks at 1799 and 2315 cm^−1^ are attributed to C=O and C–O, respectively. O–H peak also appeared at ~ 3500 cm^−1^ with a relative intensity of 2.1 compared to C=C in agreement with previous reports^11,28^. As shown, the peak intensity of the O–H functional group increases for HP due to GO in the HGO. This can be attributed to O–H functional groups in GO and adsorbed moisture. In addition, there is an extra peak in the HGO spectrum at 1622 cm^−1^, corresponding to the C=C bond in GO. As shown, there is no considerable change in the spectrum after reinforcing the polymer by GO.

Raman spectroscopy was utilized to examine the GO sheets in the HGO. Carbonic materials show G and D peaks typically at ~ 1580 and ~ 1350 cm^−1^, respectively^29,30^. The G band is attributed to E_2g_ phonon, and the D peak is due to a breathing mode of κ-point phonons with A_1g_ symmetry. The D band exhibits local defects of graphene oxide and graphite platelets. Raman spectra of the GO, HP, and HGO are presented in Fig. 1b. As shown, the G and D peaks for HGO respectively are seen at ~ 1603 and ~ 1372 cm^−1^, while these are disappeared in the HP spectrum. The G (at ~ 1582) and 2D peaks (at ~ 2680 cm^−1^) are also used to determine the layer number of GO and graphene^31–33^. As shown in Fig. 1b, the 2D band of GO at 2681 cm^−1^ overlaps with the G + D band at ~ 2850 and 3000 cm^−1^ showing the existence of single and few-layer graphene^28^.

**Figure 2:**
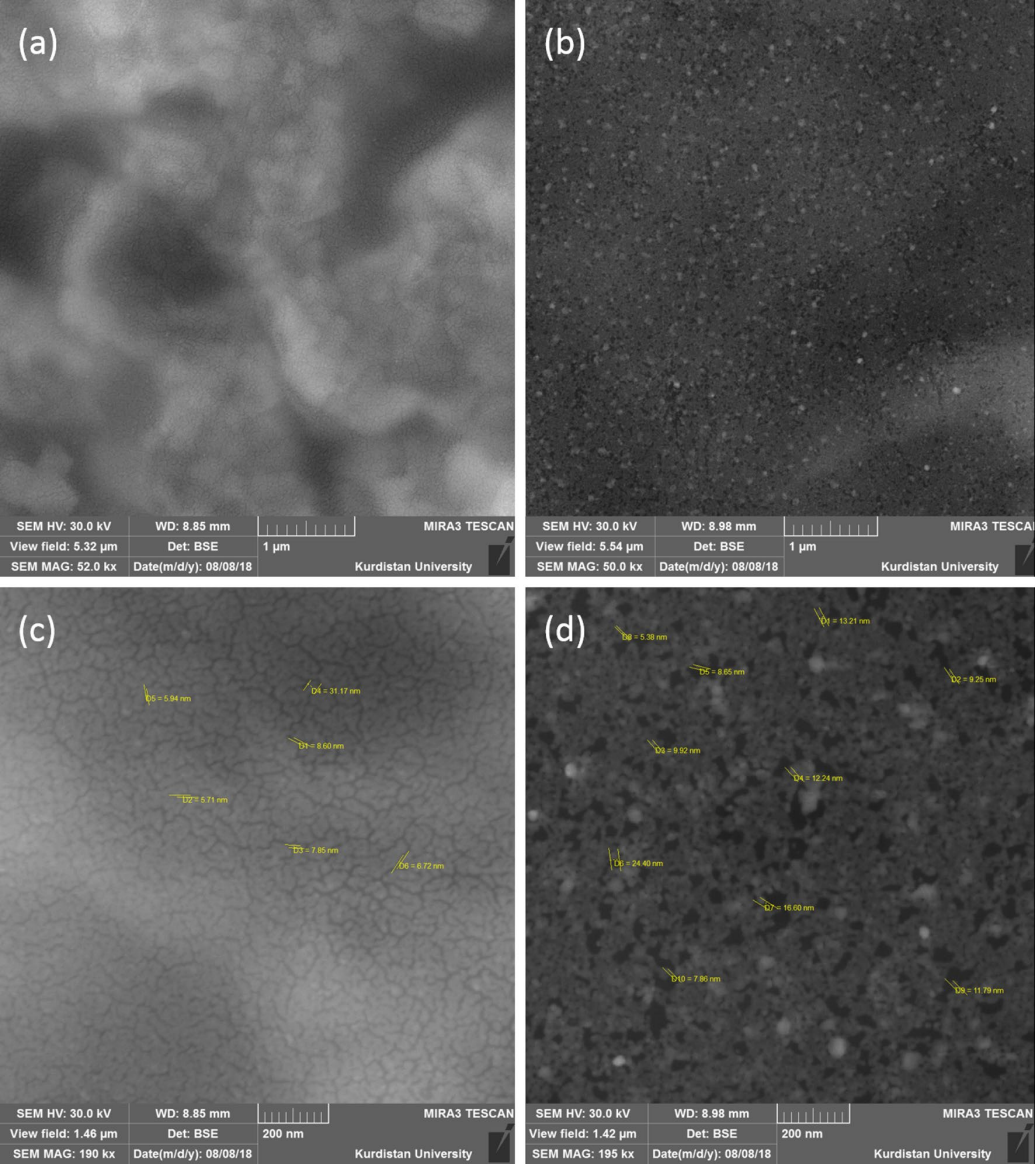
FESEM image of HP and HGO at the scale of 1 µm and 200 nm. (**a**), and (**c**) show HP surface; (**b**) and (**d**) exhibit HGO surface.

**Figure 3:**
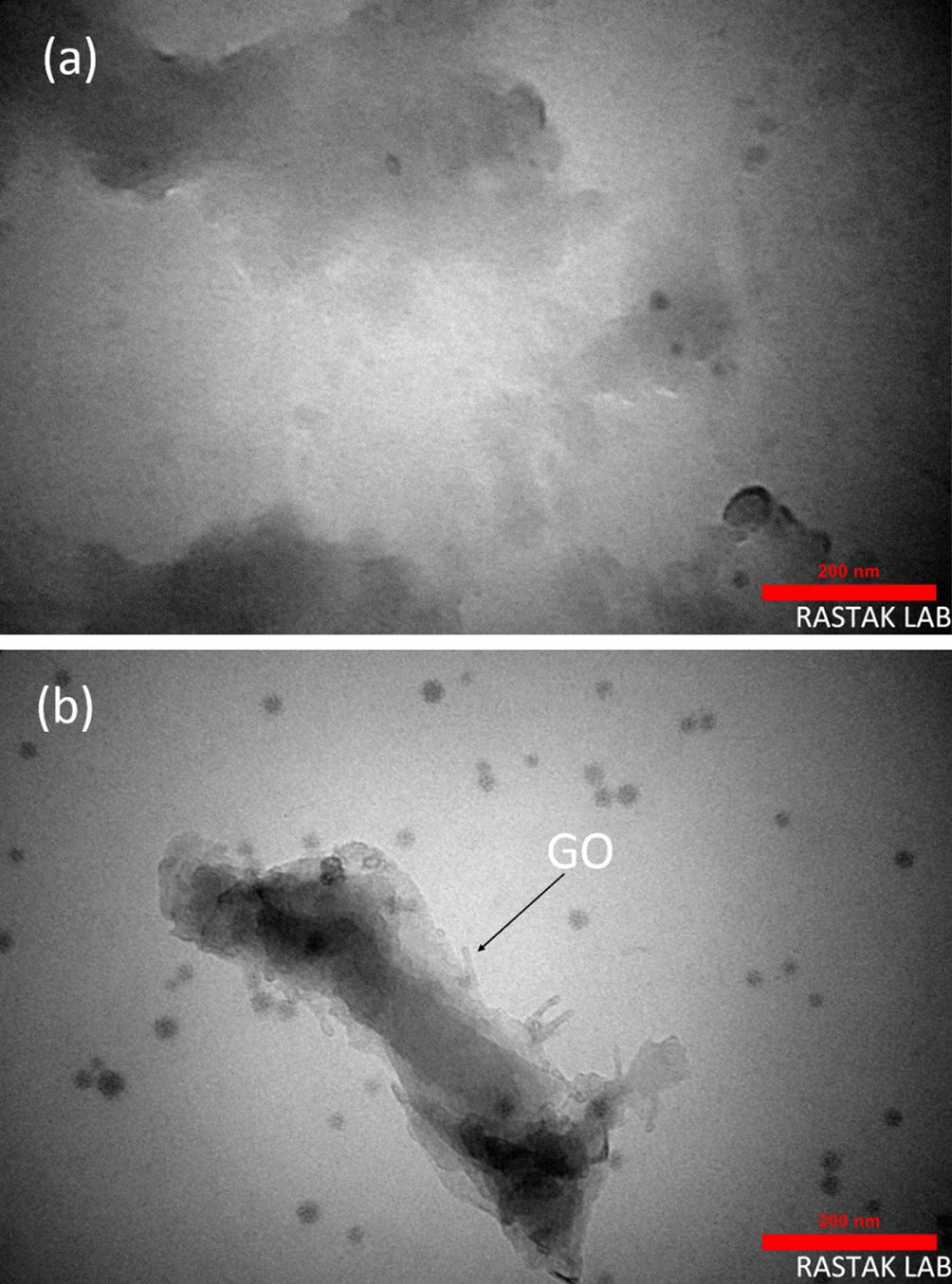
TEM image of (**a**) HP and (**b**) HGO with the scale bar of 200 nm.

The morphology of the prepared samples was investigated by FESEM. Figure 2 shows the porous surface of the prepared composite. In addition, there are some cracks on the surface of HP, while the surface of HGO has some micropores with a pore size of 5–16 nm. As shown, the porosity of the HGO seems more than the HP, which will be discussed later. In addition, the TEM image in Fig. 3 shows the presence of GO sheets inside HP and created pores in agreement with FESEM results.

**Figure 4:**
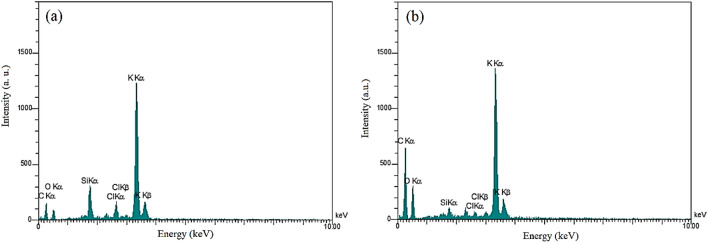
EDX Spectrum of (**a**) HP, and (**b**) HGO.

To perform elemental analysis of the samples, we used the EDX experiment. The results of EDX are shown in Fig. 4 and Table 2. As shown in Fig. 4a, about 49.04% of HP is carbon with 15.55% oxygen. However, according to Fig. 4b and Table 2 for the HGO, 65.98% of the composite is carbon, and 18.75% is oxygen. Results show that carbon and oxygen in HGO are more than HP due to GO, which agrees with FTIR results.

**Table 2:** Quantitative results obtained from the EDX spectrum of HP and HGO.

Element	Shell	Weight%		Atomic%	
		HP	HGO	HP	HGO
C	K	27.12	47.08	49.04	65.98
O	K	11.46	17.82	15.55	18.75
Si	K	4.95	0.73	3.83	0.44
Cl	K	4.01	0.78	2.45	0.37
K	K	52.46	33.60	29.13	14.46

**Figure 5:**
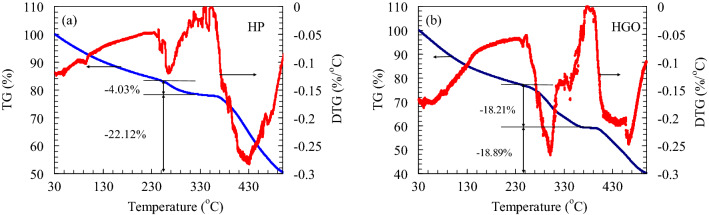
TGA and DTG for (**a**) HP and (**b**) HGO.

TGA residuum was used to quantify loaded species amount into the polymers. As shown in Fig. 5a, HP weight in TGA has two main steps. The first step (below 250 °C) is attributed to the moisture, and the second step (above 250 °C) is associated with the degradation of the hydrogels that can be several stages depending on the polymer structure. Similar behavior is observed for HGO, and as shown in Fig. 5, in the first step, moisture is released slowly at 230–310 °C with a maximum release rate at 305 °C (Fig. 5b). The different release kinetics of HP and HGO is due to the different interactions between the water molecules in HP and HGO.

**Figure 6:**
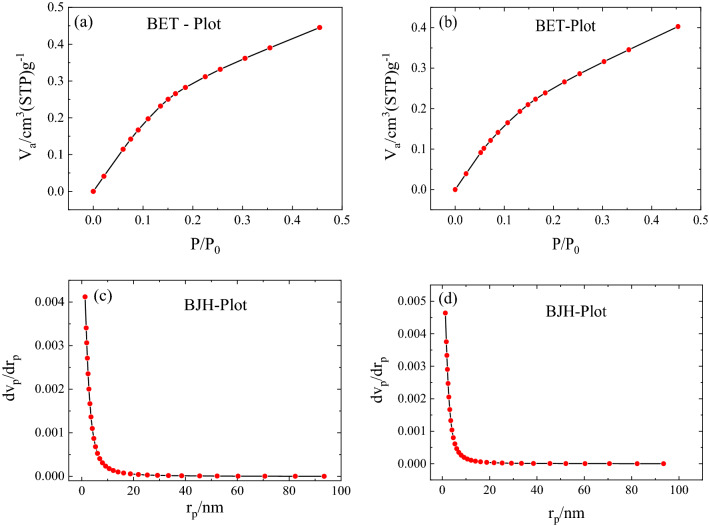
(**a**) and (**b**) show the BET plot of HP and HGO, and (**c**) and (**d**) present the BJH plot of HP and HGO, respectively. The corresponding data are presented in Table 3.

Another factor in sound absorption is the porosity of sound absorbers^12^. Polymer surface area was investigated using BET analysis from N_2_ adsorption–desorption isotherms, and results show 3.2416 m^2^ g^−1^ and 2.6716 m^2^ g^−1^ specific surface areas for HGO and HP, respectively. In addition, the average diameter of the pores in the HP is 10.274 nm, while this is 8.520 nm for HGO. The BET experiment was performed by the adsorption and desorption of nitrogen isomers at 77 K. According to BET theory, the gas adoption can be done in multi-layered form, without any reaction with adjacent molecules^34^. Typically the increasing trend at low pressure shows micropores, and the hysteresis loop at high pressure is attributed to meso/macropores. As shown in Fig. 6, the absorption and desorption graph of the HP shows type III isotherm indicating weak interaction in the first layer between absorbent and absorber^34^. However, as presented in Table 3, the HGO shows more porosity than the HP, while pore size calculations by the BJH model based on the Kelvin equation give a value of 2.58 nm for both samples^35,36^. It is known that BET calculation measures the surface areas, and results can be larger according to enhanced interactions of the gas inside the pores^37^. However, the BJH model gives the pore radius from adsorbed/desorbed volume at a given pressure according to the following formula^38^:

1 $$ln(p/{p}_{0} )=-2\gamma M/RT{r}_{k},$$

where $\gamma$ and $M$ are the surface tension of gas and the molar volume of the adsorbate, respectively. In this model, cylindrical pores are considered, and the Kelvin radius (${r}_{k}$) is taken equal to the mesopores radius minus the thickness of the adsorbed film. Since the pores in TEM and FESEM images are not cylindrical therefore one gets a smaller pore size than the mean pore size from the BET model.

**Table 3:** BET and BJH analysis details for HP and HGO.

Analysis	Sample	Special surface area (cm^2^ g^−1^)	Total volume of pore (cm^2^ g^−1^)	Mean pore diameter (BET)/pore diameter (BJH) (nm)
BET	HP	2.672	0.00686	10.27
	HGO	3.242	0.00690	8.52
BJH	HP	8.299	0.00823	2.58
	HGO	8.939	0.00945	2.58

**Figure 7:**
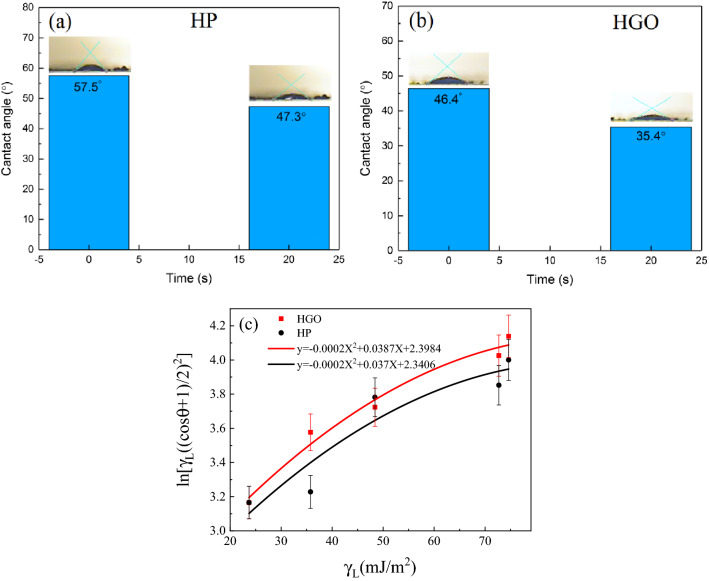
(**a**) and (**b**) the mean contact angle of water droplets on the HP and HGO immediately after dropping, and after 20 s, (**c**) The fitted curve on contact angle results of various liquids to calculate the surface energy of HP and HGO.

The contact angle of water droplets was measured on the surface of the HP and HGO. We recorded the contact angle of the droplet for at least 10 points on the surface, immediately and after 20 s. The mean contact angle of the water droplets on the HP and HGO surface is 52.4° and 40.9° at first, respectively (Fig. 7a,b). Using the Neumann method, we obtained the surface energy by the following formula:

2 $$ln\left[{\gamma }_{l}{\left(\frac{\left(\mathit{cos}\theta +1\right)}{2}\right)}^{2}\right]=\mathit{ln}\left({\gamma }_{l}\right)-\beta {\left({\gamma }_{s}-{\gamma }_{l}\right)}^{2}.$$

In Eq. (2), ${\gamma }_{l}$, ${\gamma }_{s}$, and θ represent the surface tension of the liquid, sample, and the contact angle of a droplet, respectively. To obtain the surface energy, we measured the contact angle of various liquids (including ethylene glycol, water, acetone, DMF, and H_2_O_2_) on the surface. Corresponding error bars in Fig. 7c were obtained by averaging the values at different points. By plotting $ln\left[{\gamma }_{l}{\left(\frac{\left(\mathit{cos}\theta +1\right)}{2}\right)}^{2}\right]$ versus ${\gamma }_{l}$ and fitting a second-order curve (Fig. 7c), *β* is determined as $-0.0001 {\mathrm{m}}^{4}({\mathrm{mJ})}^{-2}$, and surface energy of HP and HGO is calculated as 51.5 and 58.4 ${\mathrm{mJ m}}^{-2}$, respectively. Results confirm that the hydrophilicity of the HP increases due to GO in the polymer^39^. Adding GO as a reinforcing phase increases the surface porosity that enhances the surface roughness, leading to the increase of surface hydrophilicity according to the well-known Wenzel model^40^.

### Sound absorption

To measure the sound absorption, we used a two-microphone impedance tube with a diameter of 3 cm (BSWA model 422). The absorption coefficient, reflection, impedance, and sound admittance were measured by the impedance tube. Sound reflection measurement was carried out using the transfer function method, according to Eq. (2):

3 $$r={r}_{r}+{ir}_{i}={H}_{12}+\frac{{H}_{I}}{{H}_{R}}-{H}_{12}{e}^{i2k{x}_{1}},$$

where ${\mathrm{r}}_{\mathrm{r}}$ and ${\mathrm{r}}_{\mathrm{i}}$ are the real and imaginary components of the reflected wave, respectively. ${\mathrm{x}}_{1}$ is the sample distance from the farther microphone. ${\mathrm{H}}_{\mathrm{I}}$ and ${\mathrm{H}}_{\mathrm{R}}$ are also the transfer function for the incident and reflected wave, and ${\mathrm{H}}_{12}$ is the transfer function between the two microphones. Finally, the absorption of sound was obtained from^41^:

4 $$\alpha =1-{\left|r\right|}^{2}=1-{r}_{r}^{2}-{r}_{i}^{2}.$$

**Figure 8:**
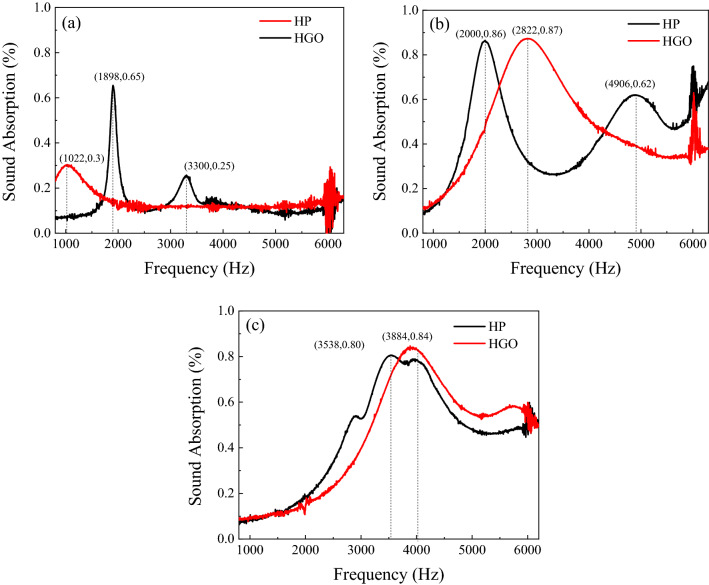
Sound absorption of (**a**) as-prepared, (**b**) after 30 min humidification, and (**c**) after 60 min humidification.

To study the humidity effect on the sound absorption of the prepared samples, we exposed them to the humidity (100% RH) for 30 and 60 min. As shown in Fig. 8a, the main absorption peak appears at 1898 Hz with an absorption coefficient of 0.65, while the second absorption peak is seen at a frequency of 3300 Hz with an absorption value of 0.25. However, for the HP, we see an absorption peak at 1022 Hz with a value of 0.3. The difference in the frequency of the absorption peak between HP and HGO can be attributed to the pores’ diameter^42^. By exposing the HGO to the humidity for 30 min, the peak shifts to the frequency of 2822 Hz with an absorption coefficient of 0.87 (Fig. 8b). However, for HP samples, the absorption peak appears at 2000 and 4906 Hz with absorption values of 0.88 and 0.62, respectively. In addition, HGO and HP after 60 min humidity exposure show the peak at 3884 and 3538 Hz, respectively (Fig. 8c). As a result, the frequency difference between HGO and bare HP decreases from 822 to 346 Hz by increasing the humidification time to 60 min.

Results in Fig. 8 show an increase in sound absorption due to humidity. Sound absorbents can reduce sound energy by converting vibrating energy into heat^43,44^. As shown in Fig. 8, sound absorption in HGO is greater than HP, and the moisture increases the intensity of sound absorption peak for HGO with the values of 0.87 and 0.84. The humidification process is repeatable, and the prepared samples can be recovered by heating for 1 h, while in the air atmosphere, it takes a month.

**Figure 9:**
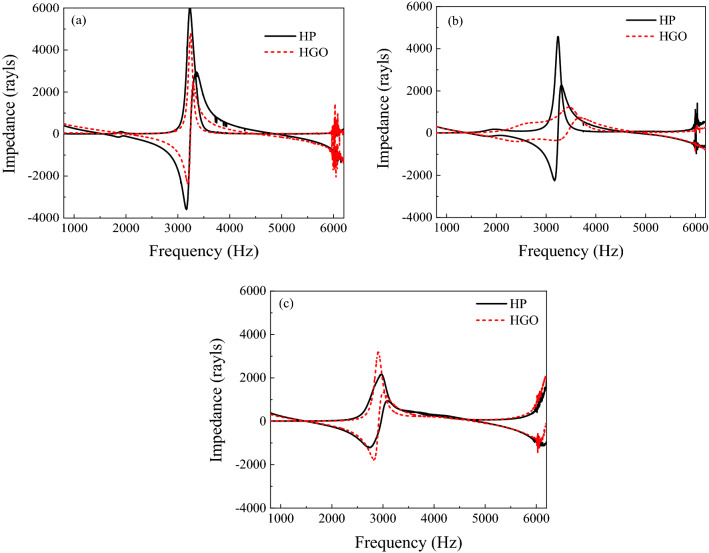
Acoustic impedance of HGO and HP after humidification for (**a**) 0, (**b**) 30, and (**c**) 60 min.

According to the Helmholtz theorem, in a resonance condition, the impedance becomes zero^44^. Figure 9a shows acoustic impedance for the HGO, which is zero in the range of 1500–2000 Hz and 3000–3500 Hz. According to the Helmholtz plot, when the acoustic impedance is zero, resonance occurs in system^11^. Figure 9a shows the maximum impedance domain in the frequency range of 2000–3000 Hz for HP and HGO, indicating the coating’s resistance to sound waves. As shown in Fig. 9b, the acoustic impedance for HGO has the highest amplitude at the frequency range of 2500–3000 Hz. Figure 9c also shows that the acoustic impedance at 2500–4500 Hz for HGO has the highest amplitude. Acoustic impedance is the ratio of applied sound pressure to the speed of the matching particle^11^. Therefore, at such a frequency of sound, one sees maximum sound absorption of the prepared samples.

## Conclusions

In summary, sound absorbers were prepared based on HP and GO, and then sound absorption was measured. We show that GO increases the surface energy of the nanocomposite as well as sound absorption. FESEM and BET results show a porous structure for HP while doping it by GO as the reinforcing phase increases porosity. The sound absorption was studied using an impedance tube in the frequency range of 1000–6000 Hz. Results show that the HGO shows two main peaks around 1898 and 3300 Hz, while HP exhibits one peak at 1022 Hz. In addition, moisture exposure tunes the sound absorption, and two absorption peaks shift to 2000 and 4906 Hz.

## Data Availability

The data presented in this study are available on request from the corresponding author.
